# “Will you need this health at all? Will you be alive?”: using the bioecological model of mass trauma to understand HIV care experiences during the war in Ukraine

**DOI:** 10.1002/jia2.26307

**Published:** 2024-07-19

**Authors:** Jill Owczarzak, Olivia Monton, Shannon Fuller, Julia Burlaka, Tetiana Kiriazova, Olga Morozova, Kostyantyn Dumchev

**Affiliations:** ^1^ Department of Health, Behavior & Society Johns Hopkins Bloomberg School of Public Health Baltimore Maryland USA; ^2^ Department of Epidemiology Johns Hopkins Bloomberg School of Public Health Baltimore Maryland USA; ^3^ Department of Mental Health Johns Hopkins Bloomberg School of Public Health Baltimore Maryland USA; ^4^ Ukrainian Institute on Public Health Policy Kyiv Ukraine; ^5^ Biological Sciences Division Department of Public Health Sciences University of Chicago Chicago Illinois USA

**Keywords:** HIV, health services accessibility, armed conflicts, Ukraine, substance use, social determinants of health

## Abstract

**Introduction:**

Russia's invasion of Ukraine in February 2022 has severely impacted the healthcare system, including the provision of HIV care. The ongoing war is a human‐caused mass trauma, a severe ecological and psychosocial disruption that greatly exceeds the coping capacity of the community. The bioecological model of mass trauma builds on Bronfenbrenner's concept of interaction between nested systems to argue that social context determines the impact of life events on the individual and how an individual responds. This paper uses the bioecological model of mass trauma to explore the impact of Russia's aggression against Ukraine and the ongoing war on HIV‐positive people who use drugs in Ukraine, a particularly vulnerable population that may be negatively affected by disruptions to social networks, healthcare infrastructure and economic conditions caused by mass trauma.

**Methods:**

Data were collected between September and November 2022. A convenience sample of 18 HIV‐positive people who use drugs were recruited from community organizations that work with people living with HIV, drug treatment programmes, and HIV clinics through direct recruitment and participant referral. A total of nine men and nine women were recruited; the age ranged from 33 to 62 years old (mean = 46.44). Participants completed a single interview that explored how the war had affected their daily lives and access to HIV care and other medical services; their relationships with healthcare providers and social workers; and medication access, supply and adherence. Data were analysed using the Framework Method for thematic analysis.

**Results:**

The war had a profound impact on the social, emotional and financial support networks of participants. Changes in social networks, coupled with limited job opportunities and rising prices, intensified financial difficulties for participants. Relocating to different regions of Ukraine, staying at somebody else's home, and losing connections with social workers impacted medication adherence and created lengthy treatment gaps. Participants also experienced a decreased supply of antiretroviral therapy, concerns about accessing medication for opioid use disorder, and overwhelming fears associated with the war, which overshadowed their HIV‐related health concerns and negatively impacted medication adherence.

**Conclusions:**

Our analysis reveals the complex impact of war on social networks and healthcare access. Maintaining support networks and competent healthcare providers will be essential amid the ongoing war.

## 1 INTRODUCTION

Russia's February 2022 invasion of Ukraine took place against a backdrop of an ongoing, 10‐year aggression that resulted in the occupation of Crimea and parts of eastern Ukraine. The 2022 invasion has resulted in over 10,000 civilian deaths and around 3.7 million people internally displaced, in addition to 13,000 killed and 1.5 million displaced since 2014 [1, 2, 3]. One year into the full‐scale invasion, 66 deliberate attacks on medical facilities have been documented, with 17 of them reporting civilian fatalities [4]. In addition, 1409 healthcare facilities were damaged and 186 have been destroyed [5, 6]. The war has severely impacted the provision of HIV services. A recent report from the Public Health Centre of the Ministry of Health of Ukraine cited that since the onset of the invasion, 38 facilities providing antiretroviral therapy (ART) were shut down, and an estimated 30% of patients on ART have experienced interruptions in medication adherence [7]. In AIDS clinics, reduced staff, overcrowding, and frequent interruptions due to air sirens have limited care providers’ ability to track patients and prescribe medications [8]. While supplies of medications for opioid use disorder (MOUD)—an essential approach to increasing HIV care engagement among people who use drugs (PWUD)—were threatened, Ukraine implemented a series of legislative and organizational steps to minimize disruption and support continued MOUD treatment among internally displaced persons [9].

**Figure 1:**
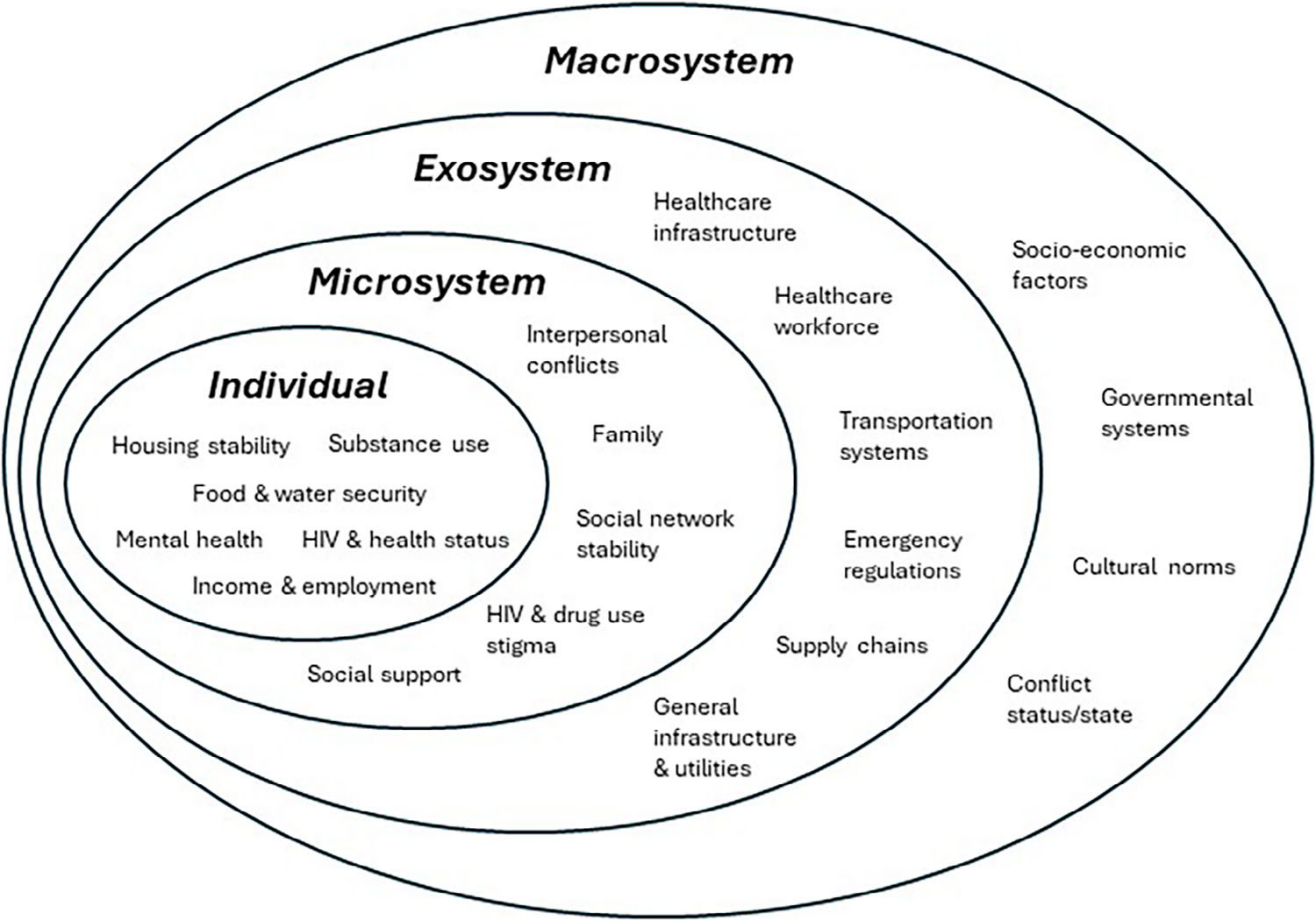
The bioecological model of mass trauma [12].

The war can be understood as a human‐caused *mass trauma*, a severe ecological and psychosocial disruption that exceeds the coping capacity of the community [10]. Mass traumas (natural disasters, terrorism, chemical accidents, wars) are dynamic events that interact with underlying social determinants of health such as poverty, fragile physical environments and illness to produce human, material, and economic losses and impacts [11]. The *bioecological model of mass trauma* builds on Bronfenbrenner's socio‐ecological model, which posits that human development is influenced by interactions within nested systems of influence, ranging from the immediate microsystem to the broader macrosystem, emphasizing the dynamic interplay between individuals and their social and environmental contexts [12]. The bioecological model of mass trauma examines the impact of large‐scale traumatic events on individuals and communities, considering the interplay between biological, psychological and environmental factors. It emphasizes the importance of understanding how these factors interact across multiple levels of influence, from individual factors and coping mechanisms to community resilience and societal responses (see Figure 1) [13, 14]. Mass traumas can exacerbate the challenges that HIV‐positive PWUD face [15]. Stress, trauma and uncertainty brought about by mass trauma can exacerbate mental health issues [16, 17, 18], further complicating HIV treatment adherence and overall wellbeing of HIV‐positive PWUD. Displaced populations or individuals seeking care in unfamiliar places may struggle to identify and access healthcare providers [19, 20]. For PWUD, disruptions in drug supply and availability of injection equipment could destabilize drug use practices, including needle sharing and engagement in high‐risk behaviours. Mass trauma can also hinder access to substance use treatment and harm reduction services [21]. Disruption of social support can exacerbate other vulnerabilities, leading to increased risk of HIV outbreaks and major setbacks in the country response to the epidemic [22].

This paper uses the bioecological model of mass trauma to explore the impact of Russia's invasion and ongoing war in Ukraine on HIV‐positive PWUD. PWUD are a key population in Ukraine's HIV epidemic, with at least 38% of all people with a new HIV diagnosis and at least 50% of all people living with HIV (PLWH) indicating injection drug use as the route of transmission [23]. In Ukraine, HIV care is delivered through specialized public funded AIDS centres and affiliates; HIV medication and treatment is provided for free to all patients registered with an AIDS centre. MOUD is delivered primarily through governmental sites although about 15% of patients receive MOUD through private clinics [9]. Understanding how the war affects this population and the strategies they use in response can inform efforts to improve post‐trauma healthcare and help them remain engaged in care.

## 2 METHODS

Data for this paper were collected as part of formative research for a larger study on HIV care retention among Ukrainian HIV‐positive PWUD being conducted in four cities (Kyiv, Odesa, Poltava and Dnipro). Data were collected between September and November 2022. A convenience sample of participants was recruited from organizations that work with PLWH, drug treatment programmes and HIV clinics. Inclusion criteria were: (1) being at least 18 years old; (2) living in one of the study cities; (3) confirmed HIV diagnosis; (4) history of injection drug use; and (5) able to consent and willing to participate. A research team member contacted potential participants, provided them with more information about the study and completed eligibility screening. All participants provided oral informed consent. Study procedures were reviewed and approved by the Institutional Review Boards at Johns Hopkins Bloomberg School of Public Health and the Ukrainian Institute on Public Health Policy.

Participants completed a single in‐person interview that explored social status and support (e.g. housing, financial situation and family relationships); substance use experiences; HIV treatment and care; relationship with healthcare providers; and access to other medical services, including drug treatment. Participants were asked about how the war affected their daily lives, access to healthcare, relationships with healthcare providers and social workers, and ART and MOUD access, supply and adherence. Interviews were conducted in either Russian or Ukrainian, based on the participant's preference. Interviews lasted around an hour and participants received the equivalent of $15 (400 UAH) as compensation.

All interviews were transcribed, translated from Russian or Ukrainian to English, and uploaded to MaxQDA 2022 for analysis [24]. Analysis began by summarizing each participant's personal background, HIV diagnosis and care history, and drug use and treatment history. Then, experiences around the war were coded and summarized using a modified framework method [25]. Codes were developed both deductively and inductively by exploring *a priori* topics that aligned with the bioecological model of mass trauma but remained open to emergent concepts. Codes included the impact of war on HIV services, family/social network, financial situation and psychological wellbeing. Codes were also developed around access to medical services, medication adherence and relationship with healthcare providers, including communication. Two coders independently coded all transcripts with the coding system, periodically checking for consistent use of codes. After each participant's transcript was coded, a summary profile was written for each participant for each code. These summaries were then analysed across participants to identify the range of experiences around receiving HIV care during the war.

## 3 RESULTS

Eighteen participants across all study cities completed in‐depth interviews (Dnipro: *n* = 4; Kyiv: *n* = 5; Odesa: *n* = 5; Poltava: *n* = 4). Participants were split evenly by sex (*n* = 9 female, *n* = 9 male); the age ranged from 33 to 62 years (mean = 46.44). Almost all participants were living with family and/or partners at the time of the interview; half of them were married, others were single (*n* = 6) or widowed (*n* = 3). Less than half (*n* = 7) were employed full‐time or part‐time, and 11 were unemployed, working occasional jobs and/or on disability status. Almost all participants had secondary education or technical training. Most had been diagnosed with HIV over 5−10 years before the interview (range: 3−30).

### 3.1 Social support and socio‐economic precarity

The start of the war prompted evacuations away from the frontlines and cities that were directly targeted by Russian ground and aerial attacks and tens of thousands of people have been drafted into the military. These movements brought significant changes to participants’ social networks with implications for social, emotional and financial support. Participants described how some friends and family who used to provide support—running errands, household chores, or financial aid—had moved and were no longer able to help. Dnipro P01, a 47‐year‐old single mother, had difficulty maintaining contact with her oldest son, who joined the army. When asked if there were people she could turn to for support, she described how her close friends had moved, although she tried to stay in touch with them by phone:

She was my social worker… I still keep in touch with her. Then I have a friend who is in Norway now, but I communicate with her too. There are a few more girlfriends. There is a neighbor who lives in Poland now. We used to be in close contact with her. But now, unfortunately, there is no such opportunity, but nevertheless we communicate with her. (Dnipro P01, female, 47 years)

Some participants also talked about the people who relied on them. These mutual obligations shaped their decisions around whether to move farther from the front lines or to stay close to care for older relatives or in areas where they had strong social ties. Odesa P04, a 51‐year‐old man, did not consider moving when the full‐scale invasion started, citing a close relationship with his elderly mother, who stayed in Odesa. They communicated often and supported each other as much as possible. When asked to elaborate on what led him to stay in Odesa, he said:

First of all, I've lived here all the time, I was born here, I have a circle of friends here, and my mother also lives here. Plus, my medicines, all my doctors ‐ everything is here, I'm tied to this place even through getting my medicines. (Odesa P04, male, 51 years)

Participants’ narratives also highlighted the complex and dynamic relationships between social/familial networks and their financial situations. Familial support was crucial in buffering against the challenging financial circumstances introduced by the war. Participants noted the dual strain of increased costs of household goods and lost employment or reduced work hours for both them and their partners. For Kyiv P04, a 38‐year‐old woman, her husband's inability to find work made it difficult for them to support each other, particularly because they had young children, and strained their relationship. Odesa P05 and his wife received disability pensions; they made ends meet but were uncomfortable. He occasionally found work as a construction contractor, but these jobs were scarce, particularly since the onset of the war. Making sure that he and his wife could support their children was a significant stressor—for example they often made personal sacrifices to ensure that their children's needs were met:

My children have shoes, clothes, food. Money is indeed running out very quickly. […] I'm already behind the shopping schedule, because I bought everything for my family, but myself, I still wear my summer shoes and my autumn jacket, roughly speaking. The main thing is to have enough money for the kids. As for myself, I'm used to it. We manage somehow. (Odesa P05, male, 43 years)

Dnipro P03, a 42‐year‐old man, summarized the problem succinctly: “[There is] not enough money. Especially lately, because of the war. You know, the prices have risen and there is less work. In general, money is a problem now. I'm not starving, but I'd like to have more money.” Many participants relied on cobbling together income and resources from multiple sources, such as disability pensions, humanitarian aid and food rations to make ends meet.

Although most participants could meet basic needs and secure housing independently or with family support, one participant, Odesa P03, a 31‐year‐old man, was often unhoused and could only check into hostels when he had money. He was not sure about any potential solutions because it had become increasingly difficult to find jobs in the informal labor market:

To be honest, I don't know anymore, I don't even know ‐ either to turn to God or to whom… Before the war, workers were gathered here in parks, paid 200, 300 hryvnias a day, and we went to construction sites, worked, and earned 250‐300‐350 hryvnias [7‐9 USD] a day; somehow it was easier. Now, with the outbreak of the war, there is no such work. And if there is work somewhere, then everything must be official ‐ all documents, you need an employment history, and so on. And I don't have it. (Odesa P03, male, 31 years)

### 3.2 Healthcare networks and care engagement

The war affected access to HIV services and other medical care in several ways, including changes to the individual and network of providers who helped people access and remain in care. In Ukraine, social workers play an important role in helping PLWH stay connected to care. Participants often had friendly, sustained relationships with social workers, whose phone calls and text messages helped them solve issues they encountered accessing care. As with other healthcare workers, some social workers left Ukraine at the start of the war and did not return. The loss of a social worker could have both temporary and longer‐term effects on people's ability to access care. Participants worried about the sometimes long and difficult process of finding and getting to know a new provider. At the start of the war, Kyiv P02, a 46‐year‐old woman diagnosed with HIV in 2015, became disconnected from her social worker and MOUD provider who left the region at the start of the war. She had to use her own contacts to find a new social worker.

Many participants experienced treatment gaps at the onset of the war, ranging from a few days to several months. Social workers played an important role in helping participants access medication and remain adherent throughout the war, for those who remained on treatment and for those who experienced treatment gaps. After a 1.5‐month treatment gap, Dnipro P01 connected with her social worker over text message, and her social worker motivated her to resume taking her medications:

I communicated with her by text messages, and she just did not suspect it right away. And only later, when I wrote to her, I said: “I quit the medications.” And she said: “I don't even want to hear anything. Resume taking medications again.” And somehow, I have had such a relationship with her for so many years, that's why she is already like a sister to me. She tells me: “When I come, I'll give you a hard time.” And that's all, I listened to her and started taking [medications] again. That's how it is. (Dnipro P01, female, 47 years)

Importantly, the sustained and trusting relationship with her social worker motivated the participant to return to treatment. Participants who did not experience treatment gaps also attested to the positive impact that their social workers had on their adherence during the war. At multiple time points, participants received the option from their social workers to either continue picking up medications in person at the AIDS centre or opt into the free delivery service. Participants also attested to consistent and frequent check‐ins and ongoing support with adherence throughout the war.

Participants who left their home cities at the beginning of the invasion encountered changes in their immediate social networks and surroundings that had implications for access to care and medication adherence. Several participants took up residence with relatives and acquaintances who were unaware of their HIV status, leading to concerns about disclosure. Kyiv P05, a 51‐year‐old‐male who relocated to Kolomyia (in western Ukraine) with his wife to live with acquaintances, worried about seeking out HIV care due to concerns for his privacy:

We live with my acquaintances; they don't know I have HIV status and I don't want them to know. They will notice if I'm looking for some kind of facility. I don't want everyone to know about it. (Kyiv P05, male, 51 years)

His concern about revealing his HIV status resulted in a months‐long treatment gap, with plans to re‐initiate treatment upon his return to Kyiv.

Temporary relocations could result in changes to familiar forms of support that undermined adherence. Kyiv P02, a 46‐year‐old woman, temporarily relocated to western Ukraine to live with her parents’ friends. She was away from her parents during this time, on whom she relied for support and reminders to take her medication. Before the war started, her father would ask about her HIV and associated treatment: Sometimes my father asks me: “How are you? How are the analyses? How do you take your drugs—do you skip it or not?” While she was in western Ukraine, she also lost contact with her social worker, who had left Ukraine and could no longer be reached. Through one of her contacts, she found and organized a consultation with a new social worker. He informed her that she could receive treatment in western Ukraine and offered to send her medication through the free delivery service. She declined and decided she would re‐initiate treatment when she returned to Kyiv, which she did after 2−3 months of missing treatment.

Participants’ healthcare networks could also be disrupted through the influx of new patients due to internal migration in some regions. As a result, wait times increased in many primary care clinics, creating barriers for patients’ registration with a primary care provider before seeing specialists. For example, Kyiv P02 wanted to see a gynaecologist but could not get a referral from a family doctor in Kyiv, who was not accepting any new patients. Additionally, two participants from Poltava described how the HIV hospital was closed and repurposed for military medical care. This did not impact their access to routine HIV care, which they received from a different centre, but they were concerned that if they needed in‐patient treatment or other general medical care, it may be difficult for them to access in non‐specialty hospitals based on prior experiences with discrimination based on HIV status.

### 3.3 Infrastructure and uncertainty

Changes and damage to physical infrastructure and supply chains created both actual and anticipated treatment gaps. At the start of the full‐scale war, halts in public transportation meant that people could not attend clinic appointments. Temporary interruptions in public transportation impacted participants who relied on picking up their medications in person. For Kyiv P01, a 37‐year‐old woman, these interruptions prevented her from accessing the AIDS centre and retrieving her medications at the onset of the war, ultimately leading to a 4‐month treatment gap. Another participant, Odesa P01, a 50‐year‐old woman, was unable to go to the clinic to get her medication when public transportation was stopped. However, in her case, she was able to get in contact with her social worker, who delivered medications to her and thus prevented a treatment gap:

It was impossible to go [to the clinic] in the first month of the war; it was impossible. On the 24th of February, the war began, and on the 27th, I ran out of drugs. And the [social workers] called me, they said: “Can you come to us?” I say: “How?” I almost cried. And they immediately rushed in and brought both humanitarian aid and pills to me. (Odesa P01, female, 50 years)

When public transit resumed, access challenges persisted. Participants from Kyiv mentioned air raid alarms resulting in sudden clinic closures. During anticipated air raids, social workers advised patients to stop clinic visits and opt for receiving mail‐delivered medication and rescheduling lab appointments. A 38‐year‐old woman from Kyiv, who had been living with HIV for 5 years and preferred to attend the clinic in‐person, described the impact of the frequent air raid alarms:

When the air [raid] alarm sounds, we're out, right? They kick you out of the room: “Wait outside for the alarm to go off, then come back.” And there are 50 people near the AIDS Center waiting. This air alarm can last for 3, 4, and 5 hours. But we still need to receive therapy or take tests. […] We cannot wait 2–3 hours every time. (Kyiv P04, female, 38 years)

In addition, before the full‐scale invasion started, most participants described an established schedule and routine for acquiring their medications, typically receiving a 3‐month supply of ART each time. Most participants picked up their medications in person at their local AIDS centre, while others opted for a free mail delivery service, which was operationalized during the COVID‐19 pandemic. At the onset of the war, many participants noted a decrease in supply (from 3 to 1 month), which eventually stabilized.

Participants who were on MOUD worried about continued access at the onset of the war. Dnipro P02, a 45‐year‐old man diagnosed with HIV in 2001, who had been on methadone maintenance therapy for 10 years, stated that he was very worried about potential gaps in MOUD when the conflict started. After hearing stories of methadone factories being bombed, he actively looked for other programmes in surrounding cities and even abroad. Luckily, he did not have any issues with access throughout wartime. Though he has had treatment gaps in ART in the past, he seemed to be more concerned about the consequences of not having access to MOUD:

You know, to be honest, if I had to choose between ART and OST [opioid substitution therapy], I would probably choose OST so that I have access to the OST, and ART is in second place. No ART, of course, also worries me. I can't say that I don't give a damn if I have it or not. But, first of all, I need OST. (Dnipro P02, male, 45 years)

For some, concerns about continued access to MOUD stemmed from stories of the 2014 Russian occupation of Crimea and its impact on drug treatment programmes. Odesa P04, a 51‐year‐old man living with HIV since 1997, said that he had been working with his doctor to gradually reduce his dose of methadone in case there are interruptions to MOUD programmes. He recalled the experience in Crimea:

I watched on TV, in 2014, when they occupied Crimea, there was a [MOUD] program there too. They showed on TV how they asked Putin not to interrupt their program. […] They say many people have died; many have left Crimea. Here in Odesa, we had a few people who recovered here and started taking medication. (Odesa P04, male, 51 years)

Kyiv P02 moved from Kyiv to Lviv (in western Ukraine) at the onset of the war. She had similar worries about MOUD access but was able to transfer from a Kyiv‐based programme to a Lviv‐based programme after learning how to through social media. As a result, she had no MOUD treatment gaps. However, she did not transfer to a new HIV care facility to continue ART and instead waited until her return to Kyiv to resume treatment. She also expressed greater concern for missing a methadone dose compared to an ART dose, because she would feel a missing methadone dose immediately.

Fear about the war also impacted medication adherence. Participants often described feeling overwhelmed and distracted, particularly at the onset of the full‐scale invasion. Having a supply of ART did not always guarantee that participants continued to adhere to medication regimens. Kyiv P02 explained that worry and stress around the war displaced her worries about her own HIV‐related health:

Because there was a fear of war, I guess it was somehow bigger than those [HIV‐related] fears, I didn't feel that fear… At first, the war came to the fore, and only later – my health. Because you did not understand ‐ will you need this health at all? Will you be alive or not? (Kyiv P02, female, 46 years)

## 4 DISCUSSION

Our analysis illustrates the intersectional and multifaceted traumas and stressors that resulted from war, whose cascading effects disrupted participants’ social networks and healthcare access for PLWH, especially those who use drugs. Although participants were mostly able to make ends meet after the start of the invasion, their social networks were under considerable strain. They had to draw on multiple social network resources to cobble together a living. Participants and their healthcare providers described pragmatic innovations to sustain access to healthcare and essential medications. These innovations, such as mail‐delivered medications and extended medication supplies to decrease the need for clinic visits, were tremendously beneficial to participants and allowed them to either stay engaged or quickly reconnect with care providers after a brief disruption. Not all participants knew how to contact providers during the early phases of the full‐scale invasion and others were not aware of the changes to medication prescribing and delivery practices. However, social media and other online forums provided crucial information channels for participants to quickly learn about changes. Supporting both providers and clients to use these forms of communication can be important tools to keep people engaged in care. While the interviews primarily focused on access to HIV care and substance use treatment, some participants noted that they were unable to access other types of necessary care, such as general primary care.

The bioecological model of mass trauma suggests that it is necessary to look beyond individual‐level effects and responses and consider the interconnections between individuals, systems and communities to understand the immediate and long‐term effects of large‐scale disasters and catastrophes [14]. For HIV‐positive PWUD, specific attention must be paid to the unique factors that affect their ability to access and remain in care, including MOUD [26]. Participants in this study described how they mitigated negative outcomes following exposure to traumatic events, including drawing on their social networks for support. However, research among PWUD has demonstrated that this population may have less access to sources of financial and other forms of support due to stigma, strained family relationships and reduced opportunities for employment [27, 28, 29]. For PLWH, a lack of supportive networks can also undermine HIV care engagement [30, 31]. As the war continues, the extent to which social networks can remain a source of financial and other forms of support is unknown. Moreover, HIV‐positive PWUD continue to face stigmatizing attitudes and behaviours in healthcare settings [32] and require experienced, empathetic and accessible providers to remain in care. In the context of strain on the healthcare workforce, exacerbated by lingering effects of the COVID‐19 pandemic, how HIV‐positive PWUD find and maintain relationships with competent providers will be an important component of understanding the long‐term effects of the war.

Ongoing war threatens further disruptions that can undermine resiliency at multiple levels. For example, conflict in the Tigray region of Ethiopia resulted in a significant loss to follow‐up of patients with chronic disease, including HIV [33]. Their experiences highlight the importance of proactive efforts to engage people in care during periods of stability and strategies that foster resiliency at the individual, network and community levels [34]. At the individual level, psychosocial support can be built through individual counselling and support groups that focus on developing adaptive coping mechanisms (e.g. seeking social support, problem‐solving) and skill‐building [35, 36]. Such efforts must be grounded in best practices of a trauma‐informed approach, which prioritize physical and emotional safety, trustworthiness, peer support, individual autonomy, flexibility and a focus on strengths [37]. Healthcare facilities and healthcare leaders can draw on the insights gained over the 2 years of the full‐scale invasion, during which they adapted by redefining their service offerings, providing additional training to first responders, deploying mobile modular services to reach patients despite the obstacles presented by damaged roads and enlisting the assistance of the national community of disease specialists to bolster care delivery for vulnerable populations [38].

The war and its consequences do not occur in a vacuum and the trauma participants currently experience did not begin in February 2022. Rather, it takes place in a context informed by both the COVID‐19 pandemic and Russia's 2014 invasion of Ukraine. As others have noted, COVID‐19 ushered in positive changes to healthcare and medication delivery that benefited PLWH and people on MOUD at the start of the full‐scale war. In Ukraine, interim Ministry of Health guidance allowed less stringent criteria for existing MOUD patients (of whom 40−45% have HIV [39]) to transition to take‐home dosing (mostly 10‐day supplies) [40]. As a result, procedures were in place at the start of the war that allowed to extend the maximum supply up to 30 days, therefore, decreasing the need to travel to clinics [9]. At the same time, COVID‐19, in Ukraine and globally, created considerable strain on the healthcare workforce, including increased workload, staff shortages, mental health challenges, and quickly changing work dynamics and workflows, leading to burnout and exodus from the profession [41]. The people we interviewed experienced the consequences of these strains directly, for example with social workers and infectious disease physicians having left the country.

In addition, following the Russian invasion in 2014 and subsequent annexation of Crimea, PLWH and PWUD witnessed first‐hand what the consequences of a Russian takeover of Ukrainian territory could mean for their health and wellbeing. In Ukraine, considerable investment has been made to increase the availability and access to MOUD [42]. However, MOUD is banned in Russia, and after the annexation of Crimea in 2014, hundreds of MOUD patients immediately lost access to this medication resulting in over 100 deaths from fatal opioid overdose, suicide and other causes [43, 44]. Other MOUD patients fled Crimea for mainland Ukraine to maintain access to this life‐saving medication. Our participants feared similar outcomes if Russia claimed new territories in the current war.

This paper has several limitations. First, data were collected as part of formative work to inform intervention design. Although war was discussed in the interviews, it was not the focus of the interview. We anticipate that interviews focused solely on the war would yield richer insights into issues related to social networks and mental health, for example. While study participants tended to emphasize positive developments and proactive responses to keep people in care, research from other conflict‐affected regions has found that war can significantly decrease healthcare utilization even in areas that are not sites of active conflict [45]. In addition, the study sample was limited to individuals who stayed in Ukraine rather than those who moved abroad. The latter likely faced unique challenges, such as navigating unfamiliar bureaucracies, learning a new language and finding ways to pay for care [46, 47, 48]. Finally, although we tried to interview people who were out of care, we were unable to recruit participants who had little contact with the healthcare sector. As a result, the experiences of our participants in many ways may represent “best case scenarios” and not the full impact of the war on HIV care engagement, particularly among the most marginalized and disengaged.

## 5 CONCLUSIONS

The ongoing war following the full‐scale Russian invasion of Ukraine has severely damaged medical infrastructure and HIV care. Participants experienced significant disruptions in their physical environments, economic stability and social support systems, which impacted their access and adherence to ART and the provision of HIV services. This was compounded by psychological stress, fear, and feelings of uncertainty. Most participants we interviewed demonstrated resiliency and creativity in response to Russia's invasion of Ukraine. In contexts of mass trauma and disruption, in addition to aid to meet basic needs, strategies should be devised to create forms of social support that help people to remain engaged in care, particularly considering the potential shortages in financial and other material resources.

## COMPETING INTERESTS

The authors declare that they have no competing interests.

## AUTHORS’ CONTRIBUTIONS

JO: Conceptualization, methodology, formal analysis, writing—original draft, funding acquisition. OM: Formal analysis, data curation, writing—original draft. SF: Formal analysis, data curation, writing—original draft. JB: Formal analysis, writing—original draft. TK: Conceptualization, methodology, investigation, resources, writing, review and editing. OM: Conceptualization, writing—review and editing. KD: Conceptualization, funding acquisition, formal analysis, writing—review and editing.

## FUNDING

Funding was provided by the National Institutes on Drug Abuse Award ID: R34 DA053143.

## Data Availability

Research data are not shared.

## References

[1] 1,458,977 Internally displaced persons. Kyiv; 2020. [cited 2024 Jun 3]; Available from: https://www.msp.gov.ua/news/19168.html

[2] United Nations Ukraine . Civilian deaths in Ukraine war top 10,000, UN says [Internet]. 2023. [cited 2024 Jun 3]; Available from: https://ukraine.un.org/en/253322‐civilian‐deaths‐ukraine‐war‐top‐10000‐un‐says

[3] Ukraine: summary of the humanitarian needs and response plan and the regional refugee response plan [Internet]. 2024. [cited 2024 Jun 3]; Available from: https://reporting.unhcr.org/operational/operations/ukraine

[4] War crimes watch Ukraine [Internet]. 2022. [cited 2024 Jun 3]; Available from: https://www.pbs.org/wgbh/frontline/interactive/ap‐russia‐war‐crimes‐ukraine/

[5] France24 . Ukraine says over 1,200 health facilities damaged since start of war [Internet]. 2023. [cited 2024 Jun 3]; Available from: https://www.france24.com/en/europe/20230212‐live‐russian‐paramilitary‐group‐wagner‐claims‐to‐have‐taken‐area‐near‐bakhmut

[6] Barten DG , Tin D , Granholm F , Rusnak D , van Osch F , Ciottone G . Attacks on Ukrainian healthcare facilities during the first year of the full‐scale Russian invasion of Ukraine. Confl Health. 2023;17(1):57. 38066621 10.1186/s13031-023-00557-2 PMC10704854

[7] National response of HIV, TB, VH and SMT programs to full‐scale Russian invasion [Internet]. Kyiv, Ukraine; 2022. [cited 2024 Jun 3]; Available from: https://www.phc.org.ua/sites/default/files/users/user92/Report_eng_final_compressed_1.pdf

[8] UNAIDS . Kryvyi Rih AIDS centre continues to provide HIV services despite the war in Ukraine [Internet]. 2022. [cited 2024 Jun 3]; Available from: https://www.unaids.org/en/resources/presscentre/featurestories/2022/may/20220525_Kryvyi‐Rih‐AIDS‐centre‐Ukraine

[9] Morozova O , Ivanchuk I , Gvozdetska O , Nesterova O , Skala P , Kuzin I , et al. Treatment of opioid use disorder in Ukraine during the first year of the Russia–Ukraine war: lessons learned from the crisis. Int J Drug Policy. 2023; 117:104062. 37216758 10.1016/j.drugpo.2023.104062 PMC11328943

[10] WHO Glossary of Health Emergency and Disaster Risk Management Terminology. Geneva; 2020.

[11] Chrisman AK , Dougherty JG . Mass trauma. Child Adolesc Psychiatr Clin N Am. 2014;23(2):257–279. 24656579 10.1016/j.chc.2013.12.004

[12] Bronfenbrenner U , Ceci SJ . Nature‐nurture reconceptualized in developmental perspective: a bioecological model. Psychol Rev. 1994;101(4):568–586. 7984707 10.1037/0033-295x.101.4.568

[13] Mao W , Agyapong VIO . The role of social determinants in mental health and resilience after disasters: implications for public health policy and practice. Front Public Health. 2021;9:115. 10.3389/fpubh.2021.658528 PMC8170026 34095062

[14] Hoffman MA , Kruczek T . A bioecological model of mass trauma. Couns Psychol. 2011;39(8):1087–1127.

[15] Karagodina O , Kovtun O , Filippovych M , Neduzhko O . Qualitative study of barriers and facilitators to HIV detection and treatment among women who inject drugs during the war against Ukraine. AIDS Res Ther. 2023;20(1):80. 37957687 10.1186/s12981-023-00578-0 PMC10644534

[16] Jack H , Reese Masterson A , Khoshnood K . Violent conflict and opiate use in low and middle‐income countries: a systematic review. Int J Drug Policy. 2014;25(2):196–203. 24332455 10.1016/j.drugpo.2013.11.003

[17] Weaver H , Roberts B . Drinking and displacement: a systematic review of the influence of forced displacement on harmful alcohol use. Subst Use Misuse. 2010;45(13):2340–2355. 20469970 10.3109/10826081003793920

[18] Ezard N . Substance use among populations displaced by conflict: a literature review. Disasters. 2012;36(3):533–557. 22066703 10.1111/j.1467-7717.2011.01261.x

[19] Ruiz‐Rodríguez M , Wirtz VJ , Idrovo AJ , Angulo ML . Access to medicines among internally displaced and non‐displaced people in urban areas in Colombia. Cad Saude Publica. 2012;28(12):2245–2256. 23288058 10.1590/s0102-311x2012001400004

[20] Rajbangshi PR , Nambiars D , Srivastava A . “We wish to have good medical care”: findings from a qualitative study on reproductive and maternal health of internally displaced women in India. Sex Reprod Health Matters. 2022;29(2):26–8282. 10.1080/26410397.2022.2059324 PMC9067944 35486074

[21] Greene MC , Kane JC , Khoshnood K , Ventevogel P , Tol WA . Challenges and opportunities for implementation of substance misuse interventions in conflict‐affected populations. Harm Reduct J. 2018;15(1):58. 30486840 10.1186/s12954-018-0267-1 PMC6263054

[22] Vasylyeva TI , Liulchuk M , Friedman SR , Sazonova I , Faria NR , Katzourakis A , et al. Molecular epidemiology reveals the role of war in the spread of HIV in Ukraine. Proc Natl Acad Sci. 2018;115(5):1051–1106. 29339468 10.1073/pnas.1701447115 PMC5798316

[23] Dumchev K , Kornilova M , Kulchynska R , Azarskova M , Vitek C . Improved ascertainment of modes of HIV transmission in Ukraine indicates importance of drug injecting and homosexual risk. BMC Public Health. 2020;20(1):1288. 32843008 10.1186/s12889-020-09373-2 PMC7449084

[24] VERBI GmbH . MAXQDA: the art of data analysis [Internet]. Berlin; 2011. [cited 2024 Jun 3]; Available from: www.maxqda.com

[25] Gale NK , Heath G , Cameron E , Rashid S , Redwood S . Using the framework method for the analysis of qualitative data in multi‐disciplinary health research. BMC Med Res Methodol. 2013;13(1):117. 24047204 10.1186/1471-2288-13-117 PMC3848812

[26] Dubov A , Basenko A , Dymaretskyi O , Shoptaw S . Impact of the Russian invasion on opioid agonist therapy programs in Ukraine: a qualitative study. Drug Alcohol Depend. 2024;255:111069. 38159338 10.1016/j.drugalcdep.2023.111069 PMC10872541

[27] Boyd J , Richardson L , Anderson S , Kerr T , Small W , McNeil R . Transitions in income generation among marginalized people who use drugs: a qualitative study on recycling and vulnerability to violence. Int J Drug Policy. 2018;59:36–43. 29986270 10.1016/j.drugpo.2018.06.014 PMC6167137

[28] DeBeck K , Shannon K , Wood E , Li K , Montaner J , Kerr T . Income generating activities of people who inject drugs. Drug Alcohol Depend. 2007;91(1):50–56. 17561355 10.1016/j.drugalcdep.2007.05.003 PMC2047290

[29] Richardson L , Wood E , Kerr T . The impact of social, structural and physical environmental factors on transitions into employment among people who inject drugs. Soc Sci Med. 2013;76:126–133. 23157930 10.1016/j.socscimed.2012.10.015 PMC3525755

[30] Smith R , Rossetto K , Peterson BL . A meta‐analysis of disclosure of one's HIV‐positive status, stigma and social support. AIDS Care. 2008;20(10):1266–1275. 18608080 10.1080/09540120801926977

[31] Higa DH , Crepaz N , Mullins MM , Adegbite‐Johnson A , Gunn JKL , Denard C , et al. Strategies to improve HIV care outcomes for people with HIV who are out of care. AIDS. 2022;36(6):853–862. 35025818 10.1097/QAD.0000000000003172 PMC10167711

[32] Owczarzak J , Fuller S , Coyle C , Davey‐Rothwell M , Kiriazova T , Tobin K . The relationship between intersectional drug use and HIV stigma and HIV care engagement among women living with HIV in Ukraine. AIDS Behav. 2022;27(6):1914–1925. 10.1007/s10461-022-03925-w 36441406 PMC9703403

[33] Gebrehiwet TG , Abebe HT , Woldemichael A , Gebresilassie K , Tsadik M , Asgedom AA , et al. War and health care services utilization for chronic diseases in rural and semiurban areas of Tigray, Ethiopia. JAMA Netw Open. 2023;6(8):e2331745. 37651138 10.1001/jamanetworkopen.2023.31745 PMC10472195

[34] Betancourt TS , Meyers‐Ohki S , Stulac SN , Elizabeth Barrera A , Mushashi C , Beardslee WR . Nothing can defeat combined hands (Abashize hamwe ntakibananira): protective processes and resilience in Rwandan children and families affected by HIV/AIDS. Soc Sci Med. 2011;73(5):693–701. 21840634 10.1016/j.socscimed.2011.06.053 PMC3162991

[35] Verduin F , Smid GE , Wind TR , Scholte WF . In search of links between social capital, mental health and sociotherapy: a longitudinal study in Rwanda. Soc Sci Med. 2014;121:1–9. 25305460 10.1016/j.socscimed.2014.09.054

[36] Rizzi D , Ciuffo G , Landoni M , Mangiagalli M , Ionio C . Psychological and environmental factors influencing resilience among Ukrainian refugees and internally displaced persons: a systematic review of coping strategies and risk and protective factors. Front Psychol. 2023;14:1–20. 10.3389/fpsyg.2023.1266125 PMC10590896 37876848

[37] Brezing C , Ferrara M , Freudenreich O . The syndemic illness of HIV and trauma: implications for a trauma‐informed model of care. Psychosomatics. 2015;56(2):107–118. 25597836 10.1016/j.psym.2014.10.006

[38] Landre JF . Leading patient‐centric crisis preparedness in healthcare: lessons from Ukraine. Healthc Manag Forum. 2023;37(2):80–85. 10.1177/08404704231208089 37931309

[39] Mazhnaya A , Marcus R , Bojko MJ , Zelenev A , Makarenko I , Pykalo I , et al. Opioid agonist treatment and improved outcomes at each stage of the HIV treatment cascade in people who inject drugs in Ukraine. J Acquir Immune Defic Syndr. 2018;79:288–295. 30312275 10.1097/QAI.0000000000001827 PMC8215524

[40] Meteliuk A , Galvez de Leon SJ , Madden LM , Pykalo I , Fomenko T , Filippovych M , et al. Rapid transitional response to the COVID‐19 pandemic by opioid agonist treatment programs in Ukraine. J Subst Abuse Treat. 2021;121:108164. 33191004 10.1016/j.jsat.2020.108164 PMC7769928

[41] Alkhamees AA , Aljohani MS , Kalani S , Ali AM , Almatham F , Alwabili A , et al. Physician's burnout during the COVID‐19 pandemic: a systematic review and meta‐analysis. Int J Environ Res Public Health. 2023;20(5):4598. 36901612 10.3390/ijerph20054598 PMC10001574

[42] Altice FL , Bromberg DJ , Dvoriak S , Meteliuk A , Pykalo I , Islam Z , et al. Extending a lifeline to people with HIV and opioid use disorder during the war in Ukraine. Lancet Public Health. 2022;7(5):e482–e484. 35358424 10.1016/S2468-2667(22)00083-4 PMC9818031

[43] Kazatchkine M . Russia's ban on methadone for drug users in Crimea will worsen the HIV/AIDS epidemic and risk public health. BMJ. 2014;348:g3118. 24812004 10.1136/bmj.g3118

[44] Carroll JJ . Sovereign rules and rearrangements: banning methadone in occupied Crimea. Med Anthropol. 2019;38(6):508–522. 30481074 10.1080/01459740.2018.1532422 PMC6536354

[45] Gedef GM , Girma B , Andualem F , Gashaw A , Tibebu NS . Antenatal care utilization and its determinants in fragile and conflict‐affected situations in Sekota Zuria District, Northern Ethiopia, 2022: a community‐based cross‐sectional study. Midwifery. 2024;129:103906. 38101294 10.1016/j.midw.2023.103906

[46] Castañeda H , Holmes SM , Madrigal DS , Young MED , Beyeler N , Quesada J . Immigration as a social determinant of health. Annu Rev Public Health. 2015;36(1):375–392. 25494053 10.1146/annurev-publhealth-032013-182419

[47] Nikitin BM , Bromberg DJ , Madden LM , Stöver H , Teltzrow R , Altice FL . Leveraging existing provider networks in Europe to eliminate barriers to accessing opioid agonist maintenance therapies for Ukrainian refugees. PLOS Glob Public Health. 2023;3(7):e0002168. 37440470 10.1371/journal.pgph.0002168 PMC10343058

[48] Feiterna‐Sperling C , Bethke H , Hofmann J , Krüger R . Refugees from Ukraine: children and adolescents with HIV in Germany. Lancet HIV. 2023;10(2):e81–e82. 10.1016/S2352-3018(22)00398-8 36642086

